# The Traits of the Regenerative Potential of Platelet-Rich Plasma from Donors with Type 1 and Type 2 Diabetes Mellitus

**DOI:** 10.3390/ijms26167856

**Published:** 2025-08-14

**Authors:** Tatyana I. Vlasova, Ekaterina P. Brodovskaya, Konstantin S. Madonov, Darya A. Kapitanova, Anna P. Abelova, Elena N. Kovalenko, Alina E. Markina, Sergey I. Pinyaev, Olga V. Minaeva, Aleksey P. Vlasov

**Affiliations:** Federal State Budgetary Educational Institution of Higher Education, National Research Ogarev Mordovia State University, Mordovia 430005, Russia; ekbrodovskaya@gmail.com (E.P.B.);

**Keywords:** platelet-rich plasma, PRP-therapy, regenerative medicine, personalized medicine, diabetes mellitus

## Abstract

The low predictability of the effects of autologous platelet-rich plasma (PRP) in regenerative therapy for patients with type 1 and type 2 diabetes mellitus (DM) underscores the need for further research assessing the reparative effects of PRP based on the type of DM. The aim of this study was to evaluate the regenerative potential of PRP from young donors (30–40 years old) with DM1 and DM2 in vitro, specifically its effects on human dermal fibroblast cell culture. The in vitro effects of PRP from patients with type 1 and type 2 DM were investigated using a culture of human dermal fibroblasts (hTERT-HDFa) to evaluate metabolic activity, migration, proliferation of the cells, and their ability to release growth factors and exosomes. The study of the biological effects of PRP from donors with DM on hTERT-HDFa revealed a decrease in proliferative effects, an increase in prooxidant action, and toxic influences of PRP from patients, characterized by reduced metabolic activity and cell viability in culture, along with an increase in the percentage of necrosis. These effects were most pronounced in type 1 DM. The secretory response of hTERT-HDFa upon stimulation with PRP varied depending on the type of DM. Correlations indicated the differing significance of PAI-1, TGFB-1, PDGF, VEGF, and IL-6 in assessing the reparative potential across different types of DM.

## 1. Introduction

Regeneration, the inherent ability of the human body to restore its structures and functions, occurs via two primary mechanisms: physiological regeneration, encompassing self-renewal during normal homeostatic processes, and pathological regeneration, which facilitates adaptation to damaging stimuli [1]. An organism’s regenerative potential, constrained by species, population, and individual factors (e.g., age and somatic status), enables both qualitative and quantitative characterization of its regenerative capacity. Studies suggest that physiological and chronological aging contribute to stem cell senescence, resulting in diminished regenerative competence [2].

Diabetes mellitus (DM) is significantly correlated with diminished regenerative potential. Epidemiological data indicate a global prevalence exceeding 500 million individuals, with projections suggesting a continued upward trend [3]. The clinical course of DM is frequently exacerbated by the development of chronic, non-healing wounds, characterized by a high recurrence rate (40% within one year, 65% within five years), thus underscoring diminished regenerative competence [4].

Diabetes mellitus is categorized into two primary etiologic subtypes. Type 1 diabetes mellitus (DM1) arises from an absolute insulin deficiency, resulting from autoimmune-mediated destruction of pancreatic β-cells [5,6]. Conversely, type 2 diabetes mellitus (DM2) is a multifactorial disorder primarily characterized by a relative insulin deficiency stemming from impaired insulin secretion and/or insulin resistance [7]. In both DM1 and DM2, chronic hyperglycemia induces damage to endothelial cells, fibroblasts, and other critical cellular constituents implicated in regenerative processes. This resultant cellular dysfunction impairs protein synthesis, proliferation, and migration, concurrent with the activation of free radical oxidation and the suppression of antioxidant systems [8].

Despite these shared consequences, the different initial stages of DM pathogenesis demonstrably influence clinical manifestations, disease severity, and complication rates, thereby necessitating distinct therapeutic strategies [9]. However, current approaches aimed at augmenting local regenerative processes in DM1 and DM2 patients often lack specificity, neglecting the nuanced pathogenesis of the disease type. This may partially account for the observed limitations and inconsistent outcomes in clinical practice [10]. Diminished regenerative potential and impaired adaptation to damaging stimuli represent a central challenge in regenerative medicine. One modality for enhancing local regenerative potential is platelet-rich plasma (PRP) therapy. The regenerative properties of PRP are largely attributed to the supraphysiological concentration of platelets, conferring a potentiated therapeutic effect compared to whole blood. Additional benefits include its autologous origin, cost-effectiveness, and extensive clinical application. However, PRP therapy is limited by poor standardization, both clinically and in research, and high variability in therapeutic response, attributable, in part, to the impact of comorbidity. Notably, specific guidelines for PRP therapy in DM patients are lacking, despite the fact that systemic DM-associated derangements affect both the effector component (platelets) and target cells (endothelial cells, fibroblasts, keratinocytes) [11,12,13]. Presently, evidence regarding the efficacy of PRP therapy for chronic wound management in DM patients remains inconsistent; some studies demonstrate limited effectiveness in treating diabetic foot ulcers [14], while others report substantial benefits [15,16].

Given the substantial unmet need for regenerative medicine approaches in diabetic patients and the current limitations in predicting the therapeutic response to PRP therapy, further investigation is warranted to elucidate the relationship between PRP’s regenerative potential and specific diabetes mellitus types.

The objective of this study was to evaluate the regenerative potential of PRP derived from young donors (30–40 years) with DM1 and DM2 through in vitro assessment of its effects on human dermal fibroblast (HDF) cell culture.

## 2. Results

### 2.1. Quantitative Characteristics of Whole Blood and PRP from DM1 and DM2 Donors

To address the inherent challenges in standardizing PRP preparation, we adopted the classification and coding system described by Kon E. et al. [17]. This approach facilitates subsequent replication of our findings by explicitly accounting for the specific attributes of the PRP preparation employed.

The code comprises a six-digit sequence, grouped into pairs, representing parameters related to platelet composition, purity, and activation: N1N2-N3N4-N5N6. Within this schema, N1 denotes the platelet concentration in whole blood, N2 denotes the platelet concentration in the PRP product, N3 indicates the presence or absence of erythrocytes, N4 indicates the presence or absence of leukocytes, N5 signifies pre-use activation status, and N6 denotes activation via the addition of CaCl2. Applying this coding system, the PRP biopreparation used in all cases was designated 26–00-11, specifying a plasma product with a 3-fold platelet enrichment, devoid of erythrocytes and leukocytes, and subjected to a two-step activation protocol.

To adequately investigate the regenerative effects of PRP, analysis of key biomolecules relevant to regeneration is necessary. Compositional data for whole blood and PRP from DM1 and DM2 donors are presented in Table 1.

Platelet levels in whole blood and PRP were comparable between donors with DM1 and DM2. Enzyme immunoassays of PRP revealed no statistically significant differences in the concentrations of pro-inflammatory cytokines and growth factors between the DM1 and DM2 groups and the control group of healthy individuals (*p* > 0.05).

**Figure 1:**
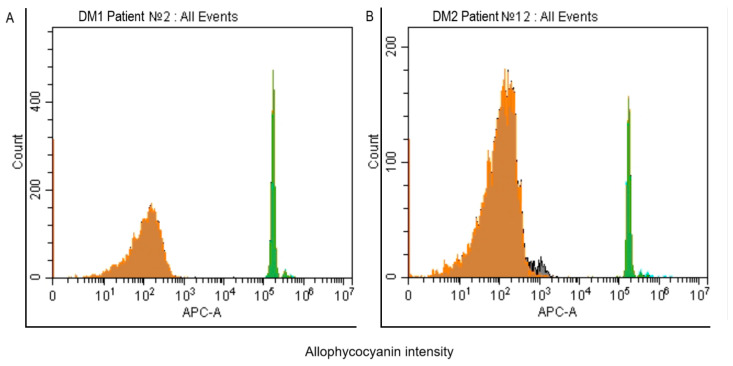
Exosome Fractions (CD9+/CD81+) in PRP Samples from DM1 and DM2 Groups (Percentage per 10,000 Events). Flow cytometry was used to identify exosomes based on forward scatter (FSC) and side scatter (SSC) properties. Each plot in Figure 1 derived from single-donor result that is representative of the respective group and indicative of the quantity of exosome in PRP donors with DM1 and DM2. (**A**). Fluorescence of exosomes in PRP samples from DM1 patient N2, captured by beads coated with anti-CD9 antibody and detected with anti-CD81 antibody (green color). (**B**). Fluorescence of exosomes in PRP samples from DM2 patient N12, captured by beads coated with anti-CD9 antibody and detected with anti-CD81 antibody (green color).

The investigation of cellular exosomes and their role in regenerative processes is an expanding field, with particular interest in exosome activity in the context of DM [18]. In this study, quantitative analysis of exosomes in the PRP of donors with DM1 and DM2 showed no statistically significant differences between the two groups (Figure 1). The median [interquartile range] number of exosomes per 10,000 recorded events was 653 [512; 1523] in the DM1 group and 597 [502; 1147] in the DM2 group, compared to 538 [489; 1028] in the control group (*p* > 0.05).

### 2.2. Assessment of Viability by MTT Test

Although much research has focused on the stimulatory effects of PRP on cell culture in the context of diabetes mellitus, limited data exist regarding the impact of PRP on fibroblast functional activity, particularly in relation to the diabetes type of the plasma donor. To investigate the influence of PRP on human dermal fibroblast biological activity, an MTT tetrazolium assay (3-(4,5-dimethylthiazol-2-yl)-2,5-diphenyltetrazolium bromide) was performed to assess cellular resistance to PRP-induced stress (Table 2).

In the control group of healthy individuals, cell viability at 24 and 48 h was comparable to that of the positive control (*p* > 0.05). However, a 28.3% decrease in viability was observed by 72 h. The DM1 group exhibited the lowest viability across all time points: at 24, 48, and 72 h, viability was significantly lower than both the positive and negative controls (*p* < 0.05). Specifically, viability was reduced by 88% and 87.6% at 24 h, 81% and 85.7% at 48 h, and 56% and 66.6% at 72 h, compared to the positive and negative controls, respectively. In the DM2 group, cell viability was significantly lower than both controls at 24 h (22% and 19.6% reduction, respectively; *p* < 0.05). At 48 h, viability in the DM2 group was significantly higher than the negative control (27% increase; *p* < 0.05) but showed a slight, albeit non-significant (*p* > 0.05), decrease compared to the positive control (4.5% reduction). By 72 h, cell viability in the DM2 group was significantly lower than both controls (67% and 75.1% reduction, respectively; *p* < 0.05). Collectively, these results indicate that PRP from both DM1 and DM2 donors negatively impacts HDF cell culture. The most significant antiproliferative effect was observed with DM1 PRP 24 h after addition, with only partial metabolic activity restoration observed after 72 h. While DM2 PRP also reduced metabolic activity at 24 h, this effect normalized by 48 h. The slight decrease in viability and metabolic activity at 72 h in the group of healthy individuals and the DM2 group is likely attributable to cell confluency and subsequent topoinhibition of growth.

### 2.3. Assessment of ROS Production

**Figure 2:**
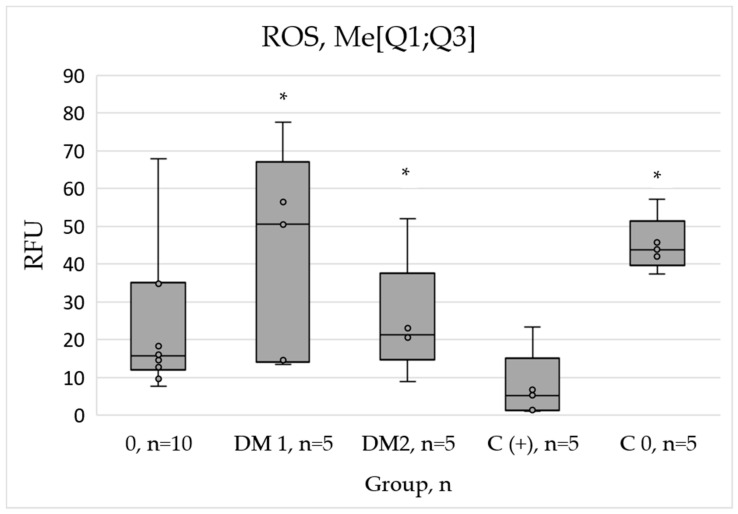
Oxidative activity in hTERT-HDFa (d220) cells following the addition of PRP from DM1 and DM2 donors and healthy individuals (group 0) * Significant difference from the positive control group (C+) at *p* < 0.05. RFU = relative fluorescence unit.

The determination of reactive oxygen species (ROS) levels in the presence of PRP from diabetic patients revealed the significant increase in oxidative activity relative to the positive control (C+). Specifically, ROS levels increased 5.2-fold and 5.1-fold in the group of healthy individuals and DM2 group, respectively, while the DM1 group exhibited a more significant increase of 11.4-fold (Figure 2).

### 2.4. Assessment of Cell Morphology and Type of Cell Death by Fluorescence Microscopy

**Figure 3:**
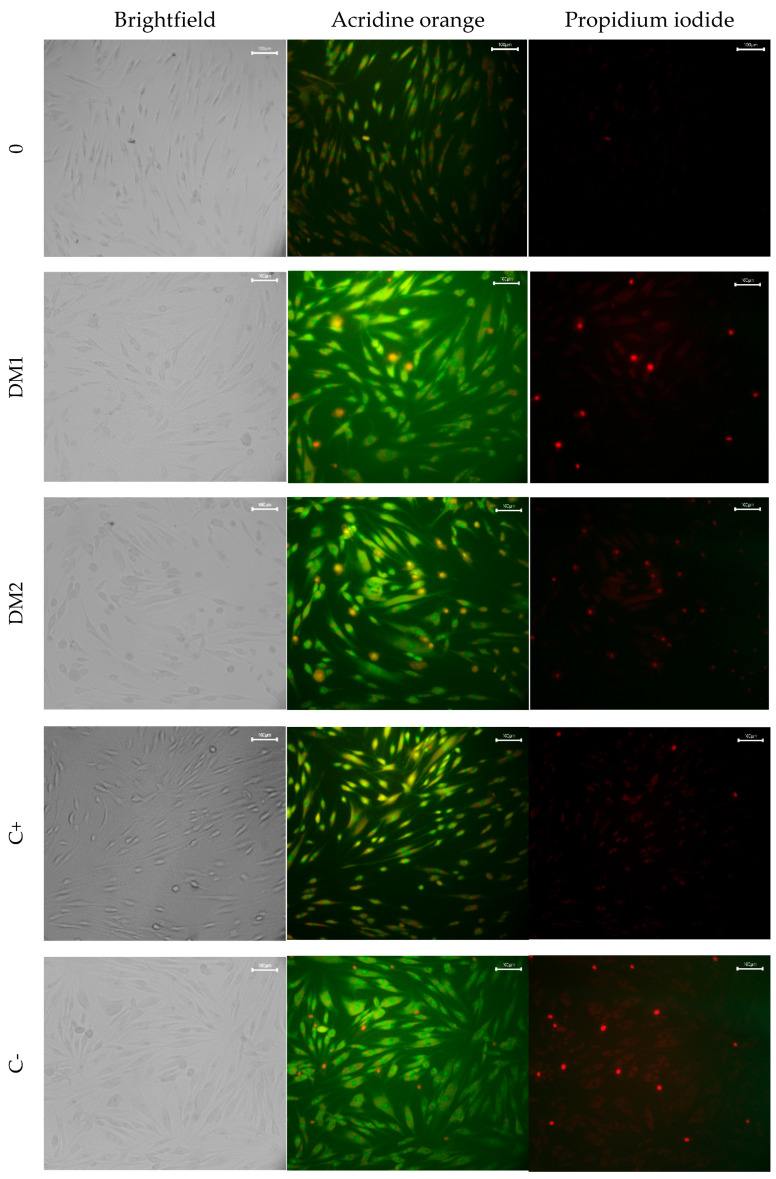
Fluorescence microscopy data of hTERT-HDFa cells, 24 h after application of PRP. Scale bar = 100 microns. AO and PI staining: AO stains the nuclei of living cells green and apoptotic cells yellow; PI penetrates only into cells with a violation of the integrity of the membrane (a marker of necrosis) and stains the nucleus red.

Fluorescence microscopy analysis (Table 3 and Figure 3) demonstrated that the proportion of viable cells in the group of healthy individuals was similar to that observed in the positive and negative control groups. However, compared to the group of healthy individuals, the DM1 and DM2 groups exhibited a comparable decrease in viable cell proportion, with reductions of 19.4% and 23.7%, respectively.

The DM2 group exhibited the highest incidence of apoptosis, showing a 171% increase (2.71-fold) compared to the healthy control group. Apoptosis rates were comparable between the remaining groups. In contrast, the DM1 group exhibited the highest incidence of necrosis. Necrosis was detected to a lesser extent in the DM2 group, was nearly absent in the positive control, present at low levels in the negative control, and completely absent in the healthy control group.

### 2.5. Assessment of Migration Activity of HDF Cell Culture

The results of the scratch assay, quantifying cell migration and proliferation, are presented in Table 4. The DM1 group demonstrated the highest proliferative and migratory activity, exhibiting a 1.31-fold greater wound closure compared to the negative control group. In contrast, the group of healthy individuals and the DM2 group showed wound closure comparable to the negative control group; however, this was 43.3% and 47.4% lower, respectively, than that observed in the positive control group.

### 2.6. Secretory Activity of Culture Cells

Enzyme immunoassay of the culture medium revealed the following patterns (Table 5).

PRP from all groups demonstrated a pro-angiogenic effect, particularly significant in the DM2 group. VEGF expression in the DM2 group was significantly elevated compared to the healthy control, positive control (C+), and negative control (C-) groups, showing increases of 60.9%, 202.2%, and 406.0%, respectively (*p* < 0.05). Similarly, PAI-1 levels in the culture medium increased following PRP stimulation, with the highest levels observed in the group of healthy individuals and the DM2 group compared to C+ and C-. PRP treatment also stimulated fibroblasts to produce more IL-6. IL-6 levels in the DM1 group were significantly elevated compared to the group of healthy individuals (34.2% increase, *p* < 0.05), and a more substantial increase was observed in the DM2 group (4.73-fold increase, *p* < 0.05). No significant differences were observed in the levels of TGFB-1, TNC, and lactate between the groups following PRP stimulation.

### 2.7. Correlation Analysis

Correlation analysis within the group of healthy individuals revealed a strong positive correlation (according to the Chaddock scale) between IL-6 levels in PRP and the percentage of scratch closure (r = 0.68). Additionally, a positive correlation was observed between PDGF levels in PRP and ROS production in the cell culture (r = 0.69), while VEGF concentration in PRP positively correlated with glycolysis (r = 0.74), indicative of active cell growth.

In the DM1 group, both PAI-1 and PDGF levels exhibited strong positive correlations with cellular metabolic activity (r = 0.72–0.87). Furthermore, a positive correlation was found between PAI-1 and TNC expression (r = 0.73) in hTERT-HDFa cell’s culture, potentially indicating an elevated risk of fibrosis during regeneration. Notably, VEGF levels in PRP were inversely correlated with the percentage of necrosis in the HDF cell culture (r = −0.80).

In the DM2 group, correlation analysis revealed a notable negative correlation between cell migration activity, as assessed by the scratch assay, and IL-6 concentration in PRP (r = −0.66). Conversely, a positive correlation was observed between cell migration activity and VEGF concentration in PRP from these donors (r = 0.72). These correlations were specific to the DM2 donor group and not observed in the other groups. In hTERT-HDFa cell’s culture, VEGF and PAI-1 concentrations exhibited inverse correlations with the percentage of necrosis (r = −0.53 and r = −0.96, respectively). Furthermore, an increase in VEGF concentration in PRP was associated with increased expression of TNC and TGFB-1. Finally, the ability of the cell culture to generate ROS was directly and strongly correlated with the initial concentration of IL-6 (r = 0.95).

## 3. Discussion

Findings from our study suggest that the levels of pro-inflammatory mediators and growth factors in PRP from healthy individuals are comparable to those observed in PRP derived from DM patients, independent of the disease type.

Evaluating the influence of DM1 and DM2 on cellular responses to PRP derived from healthy individuals, it is crucial to acknowledge that the regenerative potential of PRP from DM patients is diminished compared to PRP from healthy individuals. Our results demonstrated that the addition of PRP from DM donors significantly reduced the viability and metabolic activity of hTERT-HDFa cells, a finding consistent with prior investigations. For example, Troyanov et al. [19] similarly reported reduced growth-promoting activity of mesenchymal stem cells in vitro following stimulation with PRP from DM2 patients compared to healthy individuals. Moreover, the adverse effect of DM1 PRP was more significant and sustained (up to 72 h) than that of DM2 PRP, where a decrease was only evident during the initial 24 h, with subsequent parameters resembling those of the healthy individuals. This discrepancy may stem from elevated levels of noxious metabolites and reduced cellular production of growth factors due to significant aberrations in carbohydrate metabolism. Reinforcing this, Miroshnichenko et al. [20] demonstrated that the addition of 5% PRP from DM patients into the culture medium resulted in a greater than 40% reduction in the viability of multipotent mesenchymal stromal cells in 39% of cases.

The observed decline in metabolic activity within the initial 24 h of cell incubation with PRP from DM patients coincided with a striking elevation in ROS generation by hTERT-HDFa cell’s culture. Although a pro-oxidant effect of PRP was also detected in the healthy individuals group and the DM2 group, it was less conspicuous than that witnessed in those with DM1. Al-Mrabeh A. demonstrated that plasma cytotoxicity in DM2 patients is linked to an elevated concentration of peroxynitrite [21].

Evaluation of the magnitude and nature of cell demise following PRP addition revealed an elevated proportion of necrosis in culture added with DM1 patient PRP, while an increased number of apoptotic cells was observed in the DM2 group.

Quantification of select components of the hTERT-HDFa cells secretome following PRP stimulation yielded variable outcomes contingent on the presence of systemic diseases in the PRP donors. It is worth emphasizing that PRP addition stimulated the secretory function of dermal fibroblasts, although the intensity of this stimulation varied among the patient cohorts.

Our study revealed that the addition of PRP from DM patients prompted heightened expression of IL-6. This effect was most conspicuous with PRP derived from DM2 donors, a finding that appears to contradict the notion of reduced levels of pro-inflammatory cytokines in wounds (in vivo) during PRP therapy for chronic wounds in DM patients [22]. However, in our investigation, the level of IL-6 exhibited a positive correlation with cell migration in the context of DM2 donors, as indicated by the scratch assay results. This phenomenon is likely attributable to the regulatory function of this cytokine within the inflammatory cascade [23].

Angiogenesis, influenced by the pro-angiogenic agent VEGF, assumes a pivotal role in the restorative process [24]. In this investigation, assessment of VEGF levels in the culture medium following PRP stimulation unveiled a maximal increase in the expression of this growth factor subsequent to the addition of PRP from DM2 patients. Certain investigators have associated VEGF with the progression of micro- and macrovascular diabetic complications [25]. Notably, baseline VEGF levels in PRP from donors across the various groups were comparable. Correlation analysis illustrated a relationship between VEGF and the extent of metabolic activity in experiments employing PRP from healthy individuals. In DM1 patients, elevated VEGF content in PRP correlated with a reduction in the percentage of necrosis in hTERT-HDFa cells, substantiating the beneficial proliferative effect of this factor in DM1 [26]. In experiments involving PRP from DM2 patients, a correlation was identified between VEGF levels and TNC, as well as TGFB-1. This facet merits further scrutiny, as an elevation in profibrotic transformation markers may signify compromised regeneration and the participation of supplementary signaling pathways in paracrine regulation within the context of DM2. The acquired data are corroborated by existing scholarly works underscoring the specific significance of VEGF in the regenerative process in DM [27].

Altalhi et al. have demonstrated that the concentration of PAI-1 protease in blood plasma is elevated in individuals with DM2 as a consequence of insulin resistance, whereas findings for DM1 remain equivocal [28]. Our investigations did not reveal substantial intergroup variations in the concentration of this protease within the PRP derived from donors. The physiological function of PAI-1 secreted by fibroblasts is to safeguard intercellular matrix proteins from matrix metalloproteases, thereby facilitating wound repair [29]. Our study exhibited diminished expression of PAI-1 by hTERT-HDFa cells following PRP stimulation in DM1 patients and augmented production of this protease in DM2 patients. A decline in PAI-1 levels in the literature has been associated with compromised regeneration and a heightened propensity for fibrosis, whereas an increase in this protein has been linked to inhibition of fibrinolysis and an augmented risk of thrombotic occurrences [30].

Investigating cellular metabolite concentrations and their impact on diverse biological processes is a central goal of metabolomics. Lactate, an indicator of energy metabolism, has been extensively studied not only for its role in assessing mitochondrial function but also as a modulator of cellular processes. For instance, Horie et al. demonstrated that adipocyte-derived multipotent mesenchymal stromal cells suppress the pro-inflammatory effects of M1-polarized macrophages by secreting lactate [31]. In our study, we observed no statistically significant differences in hTERT-HDFa cells’ lactate production following PRP stimulation across the DM groups and the group of healthy individuals. However, there was a trend toward increased lactate production by the cell culture following PRP stimulation of patients in the DM1 group compared to the DM2 group. This observation warrants further mechanistic investigation to elucidate its potential significance.

## 4. Materials and Methods

### 4.1. Donors

The study cohort comprised 20 donors of European descent residing in Russia, stratified into three groups. Group 1 (Control) consisted of putatively healthy donors (*n* = 10, male/female ratio: 5/5, age range: 30–40 years, median age [interquartile range]: 36 [33; 39]). Group 2 (DM1) included donors diagnosed with type 1 diabetes mellitus (*n* = 5, male/female ratio: 1/4, age range: 30–40 years, median age [interquartile range]: 34 [31; 38]). Group 3 (DM2) encompassed donors diagnosed with type 2 diabetes mellitus (*n* = 5, male/female ratio: 1/4, age range: 30–40 years, median age [interquartile range]: 34 [31; 38]).

Inclusion criteria were as follows: (1) provision of written informed consent to participate in the study (in accordance with the ethical principles outlined in the Declaration of Helsinki); (2) a confirmed diagnosis of type 1 or type 2 diabetes mellitus, established according to the prevailing clinical guidelines promulgated by the Ministry of Health of the Russian Federation, for the DM1 and DM2 groups; and (3) achievement of controlled target blood glucose concentrations and HbA1c levels for the DM1 and DM2 groups. Exclusion criteria were as follows: (1) presence of acute or decompensated chronic illnesses; (2) history of infectious, rheumatic, autoimmune, or oncological disorders; (3) post-operative status; (4) pregnancy or lactation; and (5) routine use of antiplatelet agents, anticoagulants, or oral contraceptives.

### 4.2. PRP

Platelet-rich plasma (PRP) was generated from whole blood specimens obtained from donors. Venous blood was collected from the antecubital fossa via venipuncture, utilizing vacuum collection tubes (double-sided needle G21 0.8 × 38 mm, improve 4.5 mL tubes containing 3.8% sodium citrate anticoagulant at a 1:9 ratio). The whole blood was processed by single-stage centrifugation (270× *g* for 7 min at 22 °C) employing an ELMI Centrifuge CM-6MT centrifuge (ELMI, Riga, Latvia) equipped with a 6M.02 rotor (ELMI, Riga, Latvia). PRP was harvested from the upper plasma fraction, specifically 1 mL withdrawn from the region directly above the buffy coat. The PRP aliquots were stored in sterile 2 mL cryovials at −20 °C for a maximum duration of 1 month [32]. Platelet enumeration in both whole blood and PRP was performed using a Mindray BC-3600 hematology analyzer (Mindray, Shenzhen, China). The concentrations of vascular endothelial growth factor (VEGF) (Vector-best, Novosibirsk, Russia), transforming growth factor beta 1 (TGFB-1) (Cloud-Clone Corp, Wuhan, China), platelet-derived growth factor (PDGF) (Cloud-Clone Corp, Wuhan, China), plasminogen activator inhibitor-1 (PAI-1) (Cloud-Clone Corp, Wuhan, China), and interleukin-6 (IL-6) (Vector-best, Novosibirsk, Russia) within the PRP samples were quantified by enzyme-linked immunosorbent assay (ELISA) using commercially available immunoassay kits, adhering to the manufacturer-specified protocols.

Exosome concentrations were determined in PRP. Exosomes were isolated using a three-step centrifugation method (MF20, Domel, Suzhou, China): 500× *g* for 10 min, 2000× *g* for 10 min, and 14,000× *g* for 30 min at 4 °C. The exosomes were then stained using the commercial Exosome Isolation and Analysis Kit—Flow Cytometry, Plasma (CD9/CD81) (Abcam, Cambridge, MA, USA). This kit employs a simple immunobead assay for exosome isolation and detection, utilizing a bead-bound anti-CD9 capture antibody and a PE-conjugated anti-CD81 detection antibody. The kit offers reproducible results and can be used in conjunction with exosome immunophenotyping. Exosome concentrations were measured by flow cytometry using a CytoFLEX (Beckman Coulter, Indianapolis, IN, USA).

### 4.3. Cell Culture

Exosome quantification was performed on PRP samples. A three-step differential centrifugation method (MF20, Domel, Suzhou, China) was employed to isolate exosomes: an initial clarification spin at 500× *g* for 10 min, followed by 2000× *g* for 10 min to remove cellular debris, and a final ultracentrifugation step at 14,000× *g* for 30 min at 4 °C to pellet the exosomes. The resulting exosome-enriched fraction was then immunolabeled using the commercially available Exosome Isolation and Analysis Kit—Flow Cytometry, Plasma (CD9/CD81) (Abcam, Cambridge, MA, USA). This kit employs a simple, bead-based immunoassay for exosome capture and subsequent detection, utilizing a bead-conjugated anti-CD9 antibody for exosome binding and a phycoerythrin (PE)-conjugated anti-CD81 antibody for signal generation. This assay provides robust and reproducible results, suitable for exosome immunophenotyping studies. Quantitative exosome analysis was then conducted via flow cytometry using a CytoFLEX S instrument (Beckman Coulter, Indianapolis, IN, USA).

### 4.4. PRP Activation

Prior to introduction into the cell culture medium, PRP underwent activation via a sequential, physicochemical process. The initial step comprised a physical activation method—thawing of the previously cryopreserved PRP. This was immediately followed by a chemical activation step—the addition of a 10% CaCl2 solution (20 μl/mL) to the thawed PRP, followed by a period of incubation (22 °C, 1 h) to induce clot formation. Subsequently, the sample was centrifuged (4000× *g*, 10 min, 22 °C) to separate the activated plasma supernatant from the resulting fibrin clot.

### 4.5. Assessment of Viability and Metabolic Activity of Culture Cells

Cell Viability Assay (MTT): To assess cell viability, hTERT-HDFa fibroblasts were seeded in 96-well microplates at a density of 5000 cells per well. Following a 24-h adhesion period under standard cell culture conditions, the complete growth medium was replaced with serum-free medium supplemented with 10% donor PRP. After 24, 48, and 72 h of incubation, the medium was replaced with 100 μL of medium containing 10 μL of MTT solution (5 mg/mL) and incubated for 3.5 h to allow for formazan crystal formation within metabolically active cells. The resulting formazan crystals were then solubilized using 150 μL of DMSO. Absorbance, reflecting cell metabolic activity and thus viability, was quantified using a Varioscan Lux spectrophotometer (Thermo Fisher Scientific, Waltham, MA, USA) at 570 nm, with a reference wavelength of 650 nm [33]. Experimental results were normalized relative to the negative control, which was assigned a value of 100%.

Reactive Oxygen Species (ROS) Assay: Intracellular ROS production was quantified using the H2DCFDA assay. hTERT-HDFa cells were seeded in 96-well microplates and incubated for 24 h. Subsequently, cells were washed thrice with Hank’s Balanced Salt Solution (HBSS) to remove residual medium and incubated with 10 μM H2DCFDA (Lumiprobe, Moscow, Russia) for 45 min to allow for cellular uptake and de-esterification of the probe. Following incubation, cells were treated with 10% PRP. H2DCFDA, a non-fluorescent, cell-permeable dye, is converted to the highly fluorescent DCFDA upon oxidation by intracellular ROS. The fluorescence intensity, indicative of ROS levels, was measured using a Varioscan Lux spectrophotometer (em. 485 nm, ex. 535 nm) after 24 h of incubation [34].

Fluorescence Microscopy: Quantitative Assessment of Cell Death Mechanisms: To differentiate between live, apoptotic, and necrotic cells, hTERT-HDFa cells were seeded onto 24-well plates and cultured for 24 h under standard conditions (5% CO2 and 37 °C). The complete culture medium was then replaced with serum-free medium supplemented with 10% donor PRP. Control cells were cultured without PRP, while a positive control was supplemented with 10% FBS. After 24 h of incubation, cells were stained with acridine orange (AO) (Paneco, Moscow, Russia) at 100 µg/mL and propidium iodide (PI) (Invivogen, San Diego, CA, USA) at 100 µg/mL for 10 min in the dark [35]. Cells were visualized using a BM35FXT fluorescent inverted microscope (China), and representative fields of view were captured using a Lomo MS-20 digital camera (Lomo, Saint Petersburg, Russia). Viable, apoptotic, and necrotic cells were enumerated within the captured fields using ImageJ software, version 1.53t (NIH, Bethesda, MD, USA), with a minimum of 100 total cells counted per condition. Acridine orange (AO) permeates all cells, emitting green fluorescence upon intercalation with DNA. Propidium iodide (PI), a DNA intercalating agent, is only able to enter cells with compromised membrane integrity and emits red fluorescence upon binding to DNA. In cells where both dyes are present, PI fluorescence dominates. Consequently, viable cells have a normal green nucleus; early apoptotic cells have a bright green nucleus with condensed or fragmented chromatin; late apoptotic cells have condensed and fragmented orange chromatin; and cells undergoing necrosis have a structurally normal red nucleus [36].

### 4.6. Assessment of Migration Activity of Culture Cells

The proliferative and migratory capacity of fibroblasts was evaluated using a scratch wound assay. This in vitro assay is a widely used technique for assessing cell migration, simulating wound closure by creating a cell-free zone within a confluent monolayer and monitoring the subsequent repopulation of this area by migrating cells. hTERT-HDFa cells were seeded in 24-well plates (SPL, Gyeonggi-do, Korea) at a density of 25,000 cells per well and incubated under standard cell culture conditions for 24 h to achieve confluency. A linear scratch “wound” was introduced into the cell monolayer using a 200 µL pipette tip, after which cells were treated with 10% donor PRP. Control experiments included untreated cells and cells cultured in medium supplemented with 10% FBS. The scratch area was visualized using an inverted microscope (Micromed, Saint Petersburg, Russia) and images were captured with a Toupcam camera (Touptek, Hangzhou, China) at time points 0 h and 24 h post-scratch. The area of the residual wound was quantified at each time point using ImageJ software, version 1.53t (NIH, Bethesda, MD, USA). The percentage of wound closure, reflecting the combined effects of cell proliferation and migration, was calculated using the following formula within the Fiji Image program:

Defect closure (%) = [(Initial Wound Area (μm^2^) − Wound Area at 24 h (μm^2^))/Initial Wound Area (μm^2^)] × 100% [37].

### 4.7. Assessment of Secretory Activity of Culture Cells

Dermal fibroblasts were seeded into 24-well plates (SPL, Gyeonggi-do, Korea) and incubated under standard cell culture conditions for 24 h to allow for cell adhesion and stabilization. Following this initial incubation, the medium was replaced with DMEM supplemented with 10% donor PRP. As controls, cells were either cultured in serum-free (0% FBS) DMEM or in DMEM containing 10% FBS, representing negative and positive controls, respectively. All cells were then incubated for an additional 24 h. Subsequently, the culture supernatants were harvested and stored at −20 °C pending further analysis. The concentrations of select secreted factors within the supernatants were quantified using commercially available ELISA kits. Specifically, lactate (Olmeks, Saint Petersburg, Russia), VEGF (Vector-best, Novosibirsk, Russia), TGFβ1 (Cloud Clone Corp., Wuhan, China), PAI-1 (Cloud Clone Corp., Wuhan, China), IL-6 (Vector-best, Novosibirsk, Russia), and TNC (Cloud Clone Corp., Wuhan, China) levels were determined according to the manufacturer-provided protocols for each ELISA kit.

### 4.8. Statistical Analysis

Statistical analyses were conducted using Stattech software, version 4.7.1 (Stattech LLC, Ekaterinburg, Russia). Prior to analysis, the distribution of all quantitative data was assessed to determine appropriate statistical methods. For data exhibiting non-normal distribution patterns, results are reported as the median (Me) with interquartile range (Q1;Q3). Intergroup comparisons of quantitative variables were performed using the non-parametric Kruskal–Wallis test, followed by post hoc pairwise comparisons using Dunn’s test incorporating Holm’s correction to adjust for multiple comparisons. Statistical significance was defined as a *p* < 0.05.

## 5. Conclusions

Our investigation explored the differential biological impact of PRP from DM patients on human dermal fibroblasts, aiming to clarify the regenerative capacity of PRP in the setting of chronic inflammatory conditions. Sustained exposure (beyond 48 h) of hTERT-HDFa cells to PRP from DM1 donors elicited a reduction in proliferative capacity, a marked pro-oxidative stress response, and cytotoxic consequences. These alterations were coupled with diminished metabolic function and cell viability, alongside an elevated proportion of necrotic cells; however, cellular migration capabilities remained unaltered. Conversely, stimulation of dermal fibroblasts with PRP from DM2 patients resulted in a transient (lasting up to 24 h) decline in proliferative and metabolic activity relative to the control, accompanied by an increased percentage of necrotic cells. However, these effects were less severe than the last one from the DM1 group. A pro-apoptotic effect was also noted following stimulation with PRP from DM2 patients.

Our investigation explored the differential biological impact of PRP from donors with DM on HDF, aiming to clarify the regenerative capacity of PRP in the setting of chronic inflammatory conditions. Sustained exposure (beyond 48 h) of hTERT-HDFa cells to PRP from DM1 donors elicited a reduction in proliferative capacity, a marked pro-oxidative stress response, and cytotoxic sequelae. These alterations were coupled with diminished metabolic function and cell viability, alongside an elevated proportion of necrotic cells; however, cellular migration capabilities remained unaltered. Conversely, stimulation of dermal fibroblasts with PRP from DM2 donors resulted in a transient (lasting up to 24 h) decline in proliferative and metabolic activity relative to the control, accompanied by an increased percentage of necrotic cells. However, these effects were less severe than those induced by PRP from DM1 patients. A pro-apoptotic effect was also noted following stimulation with PRP from DM2 donors.

Future studies should prioritize the establishment of standardized benchmarks for assessing PRP efficacy, which would facilitate wider acceptance of this promising modality in regenerative medicine and refine its utilization in specific clinical contexts. Moreover, ongoing advancements in techniques for preserving growth factor bioactivity and extending their duration of action will pave the way for the discovery of innovative strategies to optimize the regenerative potential of PRP, thereby augmenting its effectiveness across heterogeneous patient populations.

## Figures and Tables

**Table 1:** Some components of whole blood and PRP from donors with DM1 and DM2.

Indicators, Me [Q1;Q3]	0	DM1	DM2	P
Platelets (blood), N × 10^9^	239.00 [232.75; 245.25]	212.00 [204.00; 217.00]	217.00 [207.00; 220.00]	P_0-DM1_ = 0.632 P_0-DM2_ = 0.583 P_DM1-DM2_ = 0.821
Platelets (PRP), N × 10^9^	647.0 [615; 663]	623.0 [598; 654]	631.3 [547; 674]	P_0-DM1_ = 0.592 P_0-DM2_ = 0.648 P_DM1-DM2_ = 0.622
VEGF (PRP), IU/mL	248.00 [181.75; 377.25]	306.00 [299.00; 415.00]	201.00 [187.00; 294.00]	P_0-DM1_ = 0.532 P_0-DM2_ = 0.383 P_DM1-DM2_ = 0.476
PDGF (PRP), pg/mL	0.70 [0.10; 1.33]	0.63 [0.00; 1.42]	1.11 [0.28; 1.51]	P_0-DM1_ = 0.541 P_0-DM2_ = 0.290 P_DM1-DM2_ = 0.178
PAI (PRP), pg/mL	181.23 [150.72; 186.14]	123.10 [107.22; 154.73]	167.85 [165.81; 215.62]	P_0-DM1_ = 0.530 P_0-DM2_ = 0.683 P_DM1-DM2_ = 0.601
IL-6 (PRP), ng/mL	2.87 [0.73; 44.55]	3.20 [1.13; 6.41]	2.98 [0.88; 57.12]	P_0-DM1_ = 0.512 P_0-DM2_ = 0.677 P_DM1-DM2_ = 0.589

**Table 2:** The results of the MTT test in the HDF culture after the addition of PRP.

Me [Q1;Q3]	24 h.	48 h.	72 h.
0	99.50 [89.75; 108.25]	129.00 [101.50; 147.00]	95.00 [84.50; 117.00]
DM1	12.00 [11.00; 13.00]	19.00 [18.00; 78.00]	44.00 [32.00; 48.00]
DM2	78.00 [13.00; 88.00]	127.00 [126.00; 135.00]	33.00 [17.00; 86.00]
C+	97.00 [96.00; 102.25]	133.00 [124.25; 137.25}	132.50 [126.00; 134.75]
C-	98.50 [95.00; 102.00]	97.50 [89.25; 109.50]	99.00 [93.50; 101.25]
P	P_0-DM1_ = 0.014 * P_C+-DM1_ = 0.013 * P_C- -DM1_ = 0.013 *	P_0-DM1_ = 0.011 * P_C+-DM1_ = 0.006 *	P_C+-DM1_ < 0.001 * P_C+ -DM2_ = 0.003 *

* *p* < 0.05.

**Table 3:** The percentage and types of cell death in the CCF culture following the addition of PRP different group donors.

	Live Cells, %, Me [Q1;Q3]	Apoptosis, %, Me [Q1;Q3]	Necrosis, %, Me [Q1;Q3]
0	93.00 [91.00; 94.00]	7.00 [5.00; 9.00]	0.00 [0.00; 0.00]
DM1	75.00 [60.00; 84.00]	7.00 [6.00; 19.00]	16.00 [10.00; 18.00]
DM2	71.00 [70.00; 81.00]	19.00 [10.00; 24.00]	6.00 [2.00; 9.00]
C+	92.50 [88.75; 96.75]	7.00 [3.00; 10.25]	1.00 [0.75; 1.00]
C-	89.50 [88.00; 91.00]	6.50 [2.50; 10.25]	1.50 [0.00; 5.75]
P	P_0-DM1_ = 0.028 P_0-DM2_ = 0.029	P_0-DM1_ = 0.134 P_0-DM2_ = 0.067	P_0-DM1_ = 0.001 P_0-DM2_ = 0.003

**Table 4:** The results of the scratch assay.

	Percentage of Wound Closure, Me [Q1;Q3]
0	48.50 [45.00; 58.25]
DM1	62.00 [51.00; 87.00]
DM2	45.00 [40.00; 56.00]
C+	85.50 [80.25; 90.75]
C-	47.00 [42.00; 52.00]
P	P_0-DM1_ = 0.354 P_0-DM2_ = 0.893

**Table 5:** Some components of HDF culture medium after addition PRP from donors with DM1 and DM2.

Indicators, Me [Q1;Q3]	0	DM1	DM2	C+	C-	P
Lactate, mmol/L	0.33 [0.30; 0.37]	0.33 [0.31; 0.50]	0.16 [0.14; 0.18]	0.09 [0.01; 0.14]	0.56 [0.32; 1.23]	P_0-DM1_ = 0.954 P_0-DM2_ = 0.391
VEGF, IU/mL	261.00 [230.25; 375.50]	254.00 [208.00; 296.00]	420.00 [274.00; 429.00]	139.00 [90.10; 179.35]	83.00 [34.00; 102.05]	P_0-DM1_ = 0.631 P_0-DM2_ = 0.016
TGFB1, pg/mL	7.29 [4.02; 9.51]	6.32 [2.14; 7.61]	8.58 [8.58; 11.04]	0.21 [0.21; 0.21]	0.00 [0.00; 0.00]	P_0-DM1_ = 0.118 P_0-DM2_ = 0.092
PAI, pg/mL	686.18 [615.63; 752.41]	359.25 [307.35; 461.61]	617.97 [600.00; 637.53]	476.57 [476.57; 476.57]	304.07 [304.07; 304.07]	P_0-DM1_ = 0.021 P_0-DM2_ = 0.745
IL-6, ng/mL	109.10 [107.18; 132.45]	146.41 [140.85; 265.95]	515.68 [159.84; 581.72]	46.08 [46.08; 46.08]	0.97 [0.97; 0.97]	P_0-DM1_ = 0.284 P_0-DM2_ = 0.041
TNC, pg/mL	2.96 [2.87; 3.05]	3.21 [1.88; 3.40]	2.95 [2.91; 2.96]	2.98 [2.98; 2.98]	3.99 [3.99; 3.99]	P_0-DM1_ = 0.424 P_0-DM2_ = 0.690

## Data Availability

The original contributions presented in this study are included in the article. Further inquiries can be directed to the corresponding author.

## Abbreviations

The following abbreviations are used in this manuscript:

PRP	platelet-rich plasma
hTERT-HDFa	human dermal fibroblasts
FBS	fetal bovine serum
DM	diabetes mellitus
DM1	diabetes mellitus type 1
DM2	diabetes mellitus type 2
PAI-1	plasminogen activator inhibitor 1
TGFB-1	transforming growth factor β 1
PDGF	platelet derived growth factor
VEGF	vascular endothelial growth factor
IL	Interleukin
TNC	Tenascin
AO	acridine orange
PI	propidium iodide
C	Control
ROS	reactive oxygen species
RFU	relative fluorescence unit
Me [Q1;Q3]	mediana [the first quartile; the third quartile]
